# Forequarter Amputation and Resection of Ribs 1–4 for Chronic Osteomyelitis

**DOI:** 10.7759/cureus.68051

**Published:** 2024-08-28

**Authors:** Milenko T Petrovic, Anapaula Rojas, Corey O Montgomery, Matthew A Steliga

**Affiliations:** 1 Pathology, University of Arkansas for Medical Sciences, Little Rock, USA; 2 General Surgery, University of Arkansas for Medical Sciences, Little Rock, USA; 3 Orthopedics, University of Arkansas for Medical Sciences, Little Rock, USA; 4 Cardiothoracic Surgery, University of Arkansas for Medical Sciences, Little Rock, USA

**Keywords:** amputation, axillary neoplasm, sarcoma soft tissue, osteo-myelitis, interthoracoscapular amputation, forequarter amputation

## Abstract

A 78-year-old woman with a history of breast cancer, melanoma, and radiation therapy presented with worsening chronic osteomyelitis and radiation necrosis of her clavicle, scapula, and upper ribs. Despite treatment with vancomycin, she experienced significant lymphedema and near-total loss of motor function in the left upper extremity. Given the progression of the disease and diminished functionality of the limb, a forequarter amputation was determined to be the only viable option beyond supportive care. The forequarter amputation was successful, and it involved the removal of the left clavicle, scapula, ribs 1-4, and the upper extremity. Within a month, the patient regained independence in all activities of daily living, highlighting the potential for improved quality of life from surgical interventions under certain circumstances. Our case serves as a reminder that the utility of the forequarter amputation extends beyond its most common uses, such as trauma or sarcoma, and in rare cases can be an option for refractory osteomyelitis of the proximal upper extremity and chest wall.

## Introduction

Forequarter amputation is a surgical resection of the upper extremity, clavicle, and scapula that is usually performed for the treatment of trauma or malignant tumors involving the shoulder or axillary region. The first forequarter amputation (also called interthoracoscapular amputation) was described by Cuming in 1808 on a patient who sustained a gunshot wound to the axilla [1,2]. Although the procedure has been historically useful in unique cases of trauma, it has more recently been indicated for malignant neoplasms in the axilla and shoulder [3]. Currently, forequarter amputations have become increasingly rare as less invasive limb-sparing operations, chemotherapies, and radiation therapies continue to improve. However, even considering its radical nature, forequarter amputation is still a necessary procedure reserved for traumatic injuries and tumors involving key structures such as the brachial plexus and/or axillary vessels [4].

Osteomyelitis is a bacterial or fungal infection of the bone. Osteomyelitis is most commonly observed as a result of diabetic foot ulcers, but it can occur due to most open wounds or surgical procedures [5,6]. Oftentimes, osteomyelitis requires surgical intervention as it can be difficult to eradicate with medical management alone. This case demonstrates that forequarter amputation may have utility extending beyond acute trauma or bone/soft tissue sarcomas.

## Case presentation

**Figure 1:**
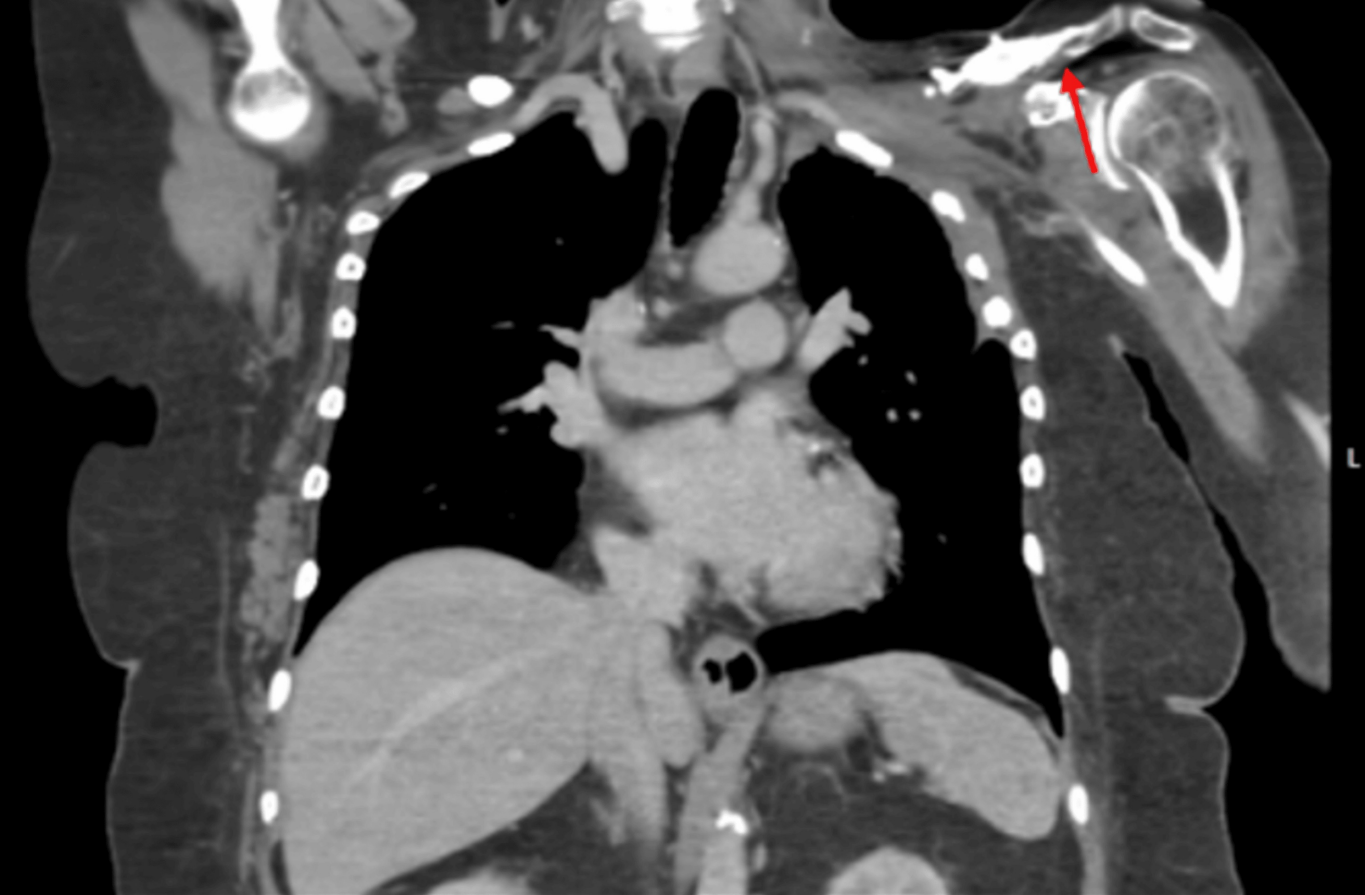
Coronal computed tomography showing soft tissue thickening and bony changes of the left scapula clavicle and humerus.

**Figure 2:**
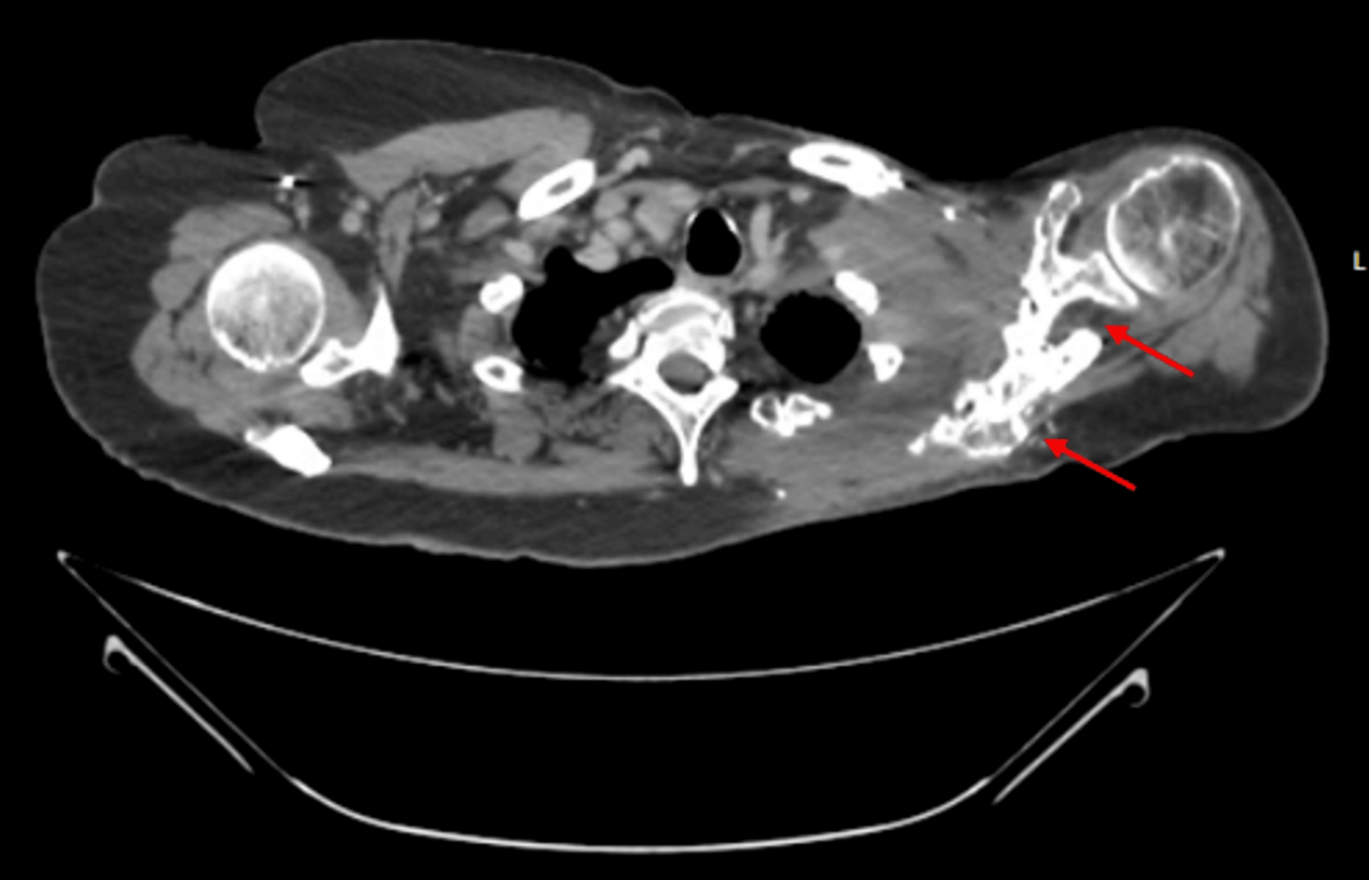
Axial computed tomography image of patient with infiltrate and sclerosis of left scapula.

A 78-year-old female with chronic osteomyelitis and radiation necrosis of the left clavicle, scapula, and rib region was referred for surgical evaluation after progression of osteomyelitis and several months of medical management with IV vancomycin HCL 1000 mg every other day. Her pertinent history includes prior breast surgery, axillary dissection, and radiation therapy for left breast cancer, as well as melanoma surgery followed by supraclavicular lymphadenectomy and further radiation therapy. Four months before the presentation, she developed an acute ischemic left limb and underwent angioplasty and stent placement. This subsequently led to gangrene at the distal tip of her fifth finger, resulting in amputation. This ischemia and amputation may have contributed to her osteomyelitis, as confirmed by methicillin-susceptible *Staphylococcus aureus* (MSSA) in blood culture after the procedure. A preoperative chest CT with contrast showed soft tissue thickening and sclerosis around the left scapula, left clavicle, humeral head, upper ribs, and surrounding soft tissue region (Figures 1-2). On physical examination, there was significant lymphedema of the affected arm, and it was grossly larger than the contralateral side. She retained sensation over the C7 dermatome; however, she had minimal to no motor function and displayed difficulty picking up a piece of paper. Her prior treatment of IV vancomycin was ineffective at addressing the osteomyelitis, and her entire upper extremity edema rendered it essentially nonfunctional. Her infection worsened with methicillin-resistant *S. aureus* (MRSA) on culture, and her creatinine slowly increased from a normal baseline to 2.5 over that time. Due to the nonfunctional status of the arm and involvement of the clavicle, scapula, humerus, and ribs, it was determined that forequarter amputation was the only option other than supportive care.

The patient was placed in a supine position. Dense radiated tissue through the infraclavicular area around the axilla and posteriorly in the area of the trapezius muscle was encountered during surgery. The pectoralis and trapezius muscles were divided with electrocautery, the scapula was elevated off the chest wall, and muscular and ligament attachments were divided with cautery. Tissue was then incised over the top of the shoulder down to the clavicle, where it was divided to its medial third using a rongeur and oscillating saw. Ribs 1-4 showed irregularity and bony destruction and were removed in a piecemeal fashion with rongeurs. The axillary artery appeared to be chronically occluded but was ligated additionally with a 4-0 Prolene suture ligature, as was the axillary vein. The left clavicle, scapula, ribs 1 through 4, and upper extremity were removed, leaving the pleura and endothoracic fascia intact. Drains were placed posteriorly along the chest wall. The residual pectoralis muscle was rotated to cover the divided neurovascular bundle stumps. Soft tissue was closed in layers; the patient was awakened and transferred to the recovery room in stable condition. The patient recovered uneventfully and was discharged home on postoperative day 7. She was back to living independently and accomplishing all activities of daily living independently, including being able to drive her riding lawn mower (with an adapted knob on the steering wheel for one-hand use) and actively working in her garden a month after discharge. After a few months of healing, she underwent fitting for an upper extremity prosthesis and further physical therapy and rehabilitation.

## Discussion

In this case, there were many indications for amputation, including failure of conservative therapy, significant impairment of upper extremity function, chronic pain, and severe lymphedema. Forequarter amputation is typically implicated in cases of trauma or malignant tumors of the upper extremity, including osteosarcomas and malignant sarcomas in the axilla. However, it has become an increasingly rare option, as only 5-10% of patients with osteosarcoma and less than 5% of patients with soft-tissue sarcomas require amputation [7]. The few reported cases of osteomyelitis near the axillary region tend to be in the clavicular region, and these were successfully treated with resection of infected bone [8,9]. In this case, we were able to observe a unique set of circumstances where a highly invasive surgery such as the forequarter amputation was required due to severe osteomyelitis.

It is important to note that osteomyelitis in the shoulder region is uncommon, and infections are far more likely to occur in the vertebrae, foot, or joint replacement site [6,10]. The etiology of this Staph aureus infection was likely multifactorial, including radiation necrosis due to prior therapies, poor microvascular circulation as an effect of radiation, bacteremia from an ischemic digit, and amputation [6,10,11].

Scarce literature is available about outcomes of treatment for osteomyelitis of the proximal upper extremity; however, Lerner et al. [12] conducted a study evaluating 109 patients with either long-bone fracture nonunion, chronic refractory osteomyelitis, or post-traumatic amputation. It was found that patients with chronic osteomyelitis had significantly worse psychologic and functional outcomes compared to the nonunion and amputation groups. In some cases, amputation has greater palliative effects compared to continued treatment and can lead to improved quality of life even for terminal diagnoses [13]. For this patient, there was necrotic bone with active infection refractory to medical management, and treatment with vancomycin was ineffective and leading to rising creatinine. This patient being able to function independently and continue performing her hobbies post-amputation would not have likely occurred without surgery. Although amputation is deforming and may be both a physical and psychological hurdle for patients to overcome, its ability to rid patients of chronic pain caused by osteomyelitis cannot be ignored. In the eyes of a patient, these quality-of-life benefits may be obscured by the radical nature of amputation, so physicians should make these benefits clear and understandable when discussing treatment options [14]. She had undergone the amputation at age 78 and continued to live independently for several years, with the last follow-up at age 88, nearly 10 years postoperatively.

## Conclusions

Forequarter amputation, albeit continually decreasing in utilization, still has its necessary uses under certain parameters, such as severe chronic osteomyelitis in the shoulder region. Because this procedure is so deforming, most surgeons and patients do not select this treatment option. Although less invasive treatments should always be considered first, this case serves as an example of the flexible applicability of the forequarter amputation, which successfully led this patient to a full recovery from a life-threatening case of osteomyelitis.
